# Once Weekly Whole-Body Electromyostimulation Enhances Muscle Quality in Men: Data of the Randomized Controlled Franconian Electromyostimulation and Golf Study

**DOI:** 10.3389/fphys.2021.700423

**Published:** 2021-07-21

**Authors:** Carina Zink-Rückel, Oliver Chaudry, Klaus Engelke, Mansour Ghasemikaram, Matthias Kohl, Michael Uder, Wolfgang Kemmler

**Affiliations:** ^1^Institute of Medical Physics, Friedrich-Alexander University Erlangen-Nürnberg (FAU), Erlangen, Germany; ^2^Medizinische Klinik III, FAU Erlangen-Nürnberg, Erlangen, Germany; ^3^Faculty Medical and Life Sciences, University of Furtwangen, Villingen-Schwenningen, Germany; ^4^Institute of Radiology, FAU Erlangen-Nürnberg and University Hospital, Erlangen, Germany

**Keywords:** whole-body electromyostimulation, muscle quality, fatty muscle infiltration, muscle tissue, men

## Abstract

Whole-body electromyostimulation (WB-EMS) is commercially advertised as a time-efficient resistance-type exercise technology. Indeed, the commercial, non-medical setting applies 20 min of WB-EMS only once a week. However, this setting conflicts with the approved scientific approach of higher training frequencies. Using data from an ongoing study on WB-EMS and golf performance as a vehicle, we evaluate the effect of once weekly WB-EMS on changes of fatty muscle infiltration, as a crucial parameter of muscle quality. Fifty-four moderately physically active male amateur golfers 18–70 years old were randomly allocated to a WB-EMS (*n* = 27) with a standard setting of once weekly 20 min and a non-WB-EMS control group (CG, *n* = 27). Intermuscular adipose tissue (IMAT) volume and intrafascial muscle tissue (MT) volume per unit of intrafascial volume as determined by magnetic resonance imaging were used to characterize muscle quality. Intention to treat analysis with multiple imputation was applied. WB-EMS was conducted at the participants’ homes; thus, the attendance rate was close to 100%. After 16 weeks of intervention, we observed increases in volume-adjusted IMAT (*p* = 0.040) and decreases in MT (*p* = 0.206) in the CG. IMAT decreased in the WB-EMS group (*p* = 0.215), while MT increased significantly (*p* = 0.032). Of importance, group difference (i.e., “effects”) for intra-group changes in volume-adjusted IMAT (effect size: *d*´ = 0.66; *p* = 0.028) and MT (*d*´ = 0.70; *p* = 0.020) was significant for both parameters. Once weekly WB-EMS application significantly affects muscle quality of the mid-thigh in moderately active, healthy men 18–70 years old.

## Introduction

**Figure 1:**
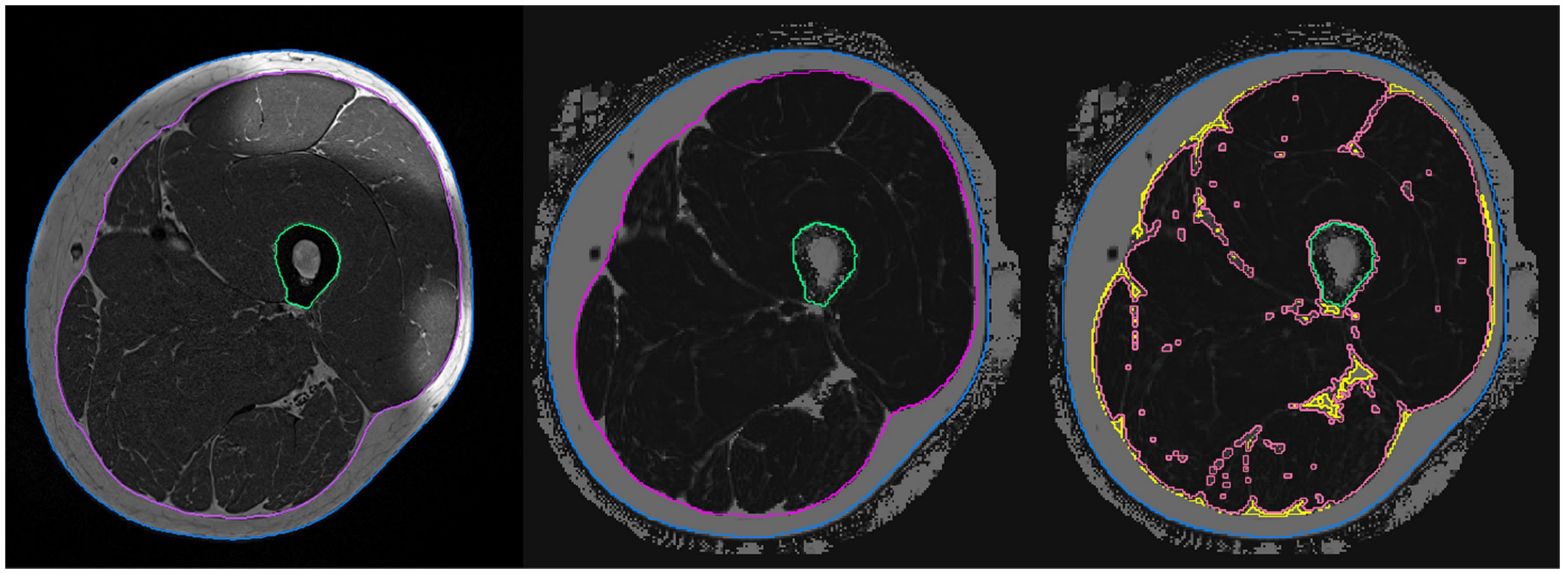
Segmentation and analysis of the magnetic resonance images. Left: T_1_-weighted image used for segmentation of the fascia (magenta), outline (blue), and femur (green). Center: Dixon image after registration of the analysis volumes. Right: Dixon image – separation into IMAT (yellow) and MT (light red) volumes.

Whole-body electromyostimulation (WB-EMS) is promoted as a time-efficient, novel exercise technology for enhancing musculoskeletal parameters in non-athletic adults. Indeed, there is considerable evidence that WB-EMS significantly affects muscle strength and mass in non-athletic cohorts (Kemmler et al., 2021). However, with few exceptions (Kemmler et al., 2016; Micke et al., 2021), all WB-EMS trials applied higher training frequencies (≥1.5 vs. 1 sessions/week) compared to the commercial, non-medical application^1^ with its rapidly growing market. Thus, the untested generalization of scientific results to the commercial setting is hardy justified. However, applying the standard setting of commercial WB-EMS in Germany (once weekly 20 min), we observed significant positive effects on maximum trunk and leg-extensor strength in a cohort of amateur golfers 18–70 years old (Zink-Rückel et al., 2021). Nevertheless, apart from this proof of principle, it is important to determine underlying mechanisms for strength changes after WB-EMS application. “Muscle quality” might be an adequate candidate for this approach. Although there is an ongoing debate as to which parameters characterize muscle quality (MQ), fatty muscle infiltration as assessed by magnetic resonance imaging (MRI) applying enhanced segmentation and automated quantification software may well be a reliable and clinically relevant morphometric predictor of MQ (Chaudry et al., 2020; Engelke et al., 2020). In the present study, we thus aimed to determine the effect of once weekly 20 min WB-EMS on fatty muscle infiltration at the mid-thigh in moderately active men 18–70 years old. In order to adequately characterize this feature, we analyzed changes in muscle tissue (MT) volume and in intermuscular adipose tissue (IMAT) volume, both normalized to changes in intrafascial volume. Briefly, IMAT is the combination of larger agglomerations of adipocytes within muscles that are visible in MR images with the perimuscular adipose tissue. Within the fascia, MT is the complement of IMAT but still contains adipocytes not visible in the MR images as well as intracellular lipids. Thus, even muscle tissue contains fat (Figure 1).

Our primary hypothesis was that once weekly WB-EMS significantly reduces both volume-adjusted IMAT (primary study outcome) and volume-adjusted MT in moderately active men compared to a non-WB-EMS control group.

## Materials and Methods

For the present evaluation, we used data from the Franconian EMS and Golf (FREMGO) study that evaluates the effect of once weekly WB-EMS on changes of golf performance, strength, and body composition (Zink-Rückel et al., 2021), including fatty muscle infiltration in a cohort of healthy amateur golfers 18–70 years old. The study was initiated and conducted by the Institute of Medical Physics, Friedrich-Alexander University Erlangen-Nürnberg (FAU), the present MRI approach was supported by the Institute of Radiology, FAU. FREMGO was approved by the FAU Ethics Committee (number 377_19b) and fully complies with the Helsinki Declaration “Ethical Principles for Medical Research Involving Human Subjects.” All study participants gave their written informed consent after having received detailed information. The FREMGO project was fully registered under ClinicalTrials.gov: NCT04264416.

### Participants

**Figure 2:**
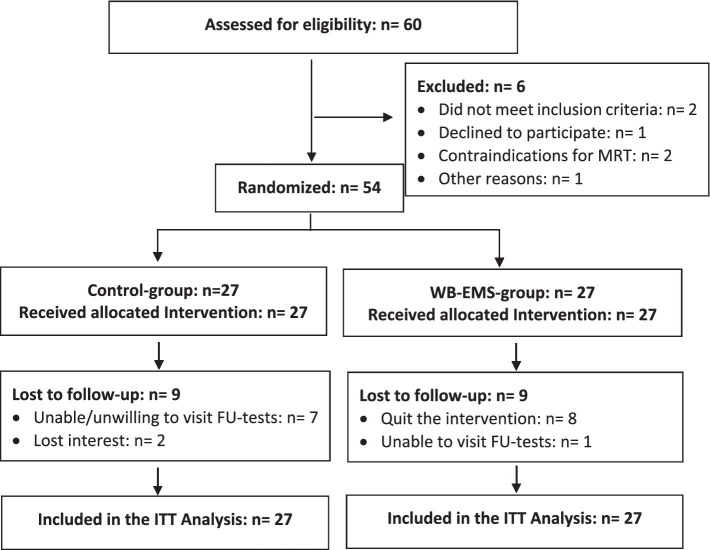
Participants flow through the FrEMGo study.

Briefly, based on personal information, information *via* social media and announcements in local golf clubs displaying the most important eligibility criteria, 60 male amateur golfers living in northern Bavaria, Germany were further assessed for eligibility. Applying the inclusion criteria: (1) men 18 to 70 years old with (2) more than 2 years’ experience in golfing and (3) a golf handicap of 54 or better, and excluding men with (1) absolute contraindication for WB-EMS (Kemmler et al., 2019), (2) contraindications for MRI (e.g., cardiac pacemakers), (3) WB-EMS application during the last 12 months, and (4) resistance exercise for more than 60 min/week during the last 12 months, 54 participants were included in the study (Figure 2).

### Randomization and Allocation

Using two strata for age, participants allocated themselves to the WB-EMS (*n* = 27) or the non-EMS control group (*n* = 27) by drawing lots placed in small opaque plastic containers (“kinder egg,” Ferrero, Italy). A research assistant not involved in the study supervised the randomization procedure. We ensured that neither the participants nor the research assistants knew the group allocation beforehand. After group allocation, the principal investigator (CZ-R) enrolled participants and informed them in detail about their duties and corresponding dos and don’ts. Of importance, due to the close personal relationships between many participants, we consider a blinding of participants (e.g., by sham WB-EMS) to be less promising. Since parallel successful blinding of treatment providers (i.e., EMS instructors) was not realistic in our setting (see below), emphasis was placed on blinding research assistants so that they were not aware of the participants’ group allocation.

### Intervention

We used WB-EMS equipment (miha bodytec^®^, Type II, Gersthofen, Germany) that enabled simultaneous, but regionally dedicated stimulation of all major muscle groups (thighs, upper arms, hip/bottom, abdomen, chest, lower back, upper back, and latissimus area) with a total stimulation area of 2,600 cm^2^. We applied a once per week 20 min standard protocol with bipolar electric current, an impulse frequency of 85 Hz, an impulse width of 350 μs with 6 s of EMS stimulation with a direct impulse boost intermitted by 4 s of rest for 16 weeks (mid-January to mid-May 2020). During the impulse phase, two sets with 6–8 reps of 8 slight movements/exercises in a standing position were performed. Exercise/impulse intensity was prescribed using the rate of perceived exertion (RPE) approach. Applying the Borg CR-10 scale, after 4 weeks of WB-EMS conditioning with lower impulse intensity, instructors encouraged participants to exercise at a RPE of “6–7” (i.e., “hard+ to very hard”). During the sessions, the impulse intensity of each electrode was adjusted every 3 min in close cooperation with participants to maintain the prescribed RPE during the session. All sessions were closely supervised with a ratio of one instructor to one participant. Here, we are strictly following the protocol applied by the vast majority of commercial, non-medical WB-EMS facilities – at least in Europe. However, for logistic reasons and finally due to the COVID-19 pandemic, the WB-EMS workouts were performed at the participants’ home or a location of their choice. Further, WB-EMS was carried out with a strict COVID-19 compliant hygiene concept. This includes strict adherence to the distance rules and wearing of medical masks. Only one instructor and one participant were allowed in the room. The equipment was cleaned and disinfected by the instructor before and after the WB-EMS application.

### Study Outcomes

Main Outcomes

- Changes in volume-adjusted IMAT of the mid-thigh as determined by MRI from baseline to 16-week follow-up.

Secondary Outcomes

- Changes in volume-adjusted muscle tissue of the mid-thigh from baseline to 16-week follow-up as determined by MRI.

Explanatory Outcomes

- Changes in IMAT volume of the mid-thigh as determined by MRI from baseline to 16-week follow-up.
- Changes in muscle tissue volume of the mid-thigh as determined by MRI from baseline to 16-week follow-up.
- Changes in intrafascial volume of the mid-thigh from baseline to 16-week follow-up as determined by MRI.
- Changes in fat fraction of muscle tissue of the mid-thigh from baseline to 16-week follow-up as determppwiz.cplined by MRI.

### Changes in Trial Outcomes After Trial Commencement

We are unable to address the primary study endpoint of FrEMGo, “golf performance” due to the COVID-19 pandemic-induced temporary closure of the golf courses in our region. However, the MRI assessments of the project performed to generate deeper mechanistic insight could be conducted as intended.

### Assessments

Prior to baseline and follow-up (FU) assessments, all participants were requested to refrain from intense physical activity and exercise 48 h pre-testing. Assessments were consistently performed at the same time of the day (±90 min), in the same order and by the same researcher using identically calibrated devices, in exactly the same setting.

Body height was assessed by a stadiometer; body mass and composition were measured using a direct segmental, multi-frequency bio-impedance analysis (DSM-BIA, InBody770, Seoul, Korea).

MRI acquisition was performed on a 3T scanner (MAGNETOM PRISMAfit, Siemens Healthineers AG, Erlangen, Germany) using an 18-channel body surface coil. Thirty slices with a thickness of 3 mm covering a total length of 9 cm were acquired. The protocol included a T1-weighted turbo spin echo and a 6-point Dixon gradient echo sequence to determine proton density fat fraction (FF; Grimm et al., 2018). Intensities in the Dixon FF images ranged from 0 to 1,000 corresponding to an FF of 0.0–100.0%. Image analysis was conducted using Medical Image Analysis Framework (FAU). Segmentation masks were generated in the T1 images and registered to the quantitative Dixon FF images as described by (Chaudry et al., 2020). This method has been successfully applied in other studies (Chaudry et al., 2020; Engelke et al., 2020; Ghasemikaram et al., 2021). Based on the histogram of the FF values of all voxels of the intra fascia volume, a threshold was determined to separate IMAT from MT. In the same volume, intrafascial fat fraction can easily be determined as the average FF value. The ratio of IMAT volume to intrafascia volume was calculated in order to adjust for changes in volume of the fascia to address elastic deformations between baseline and follow-up measurements (Figure 1).

To determine the baseline characteristics of our participants, we used questionnaires that asked for (1) demographic parameters, (2) pain frequency and intensity at the lumbar spine site, (3) diseases, limitations, injuries, and operations, (4) pharmacologic agents and dietary supplements, (5) lifestyle including nutritional habits, (6) general physical activity and exercise (Kemmler et al., 2004a,b), and (7) golf-specific aspects (i.e., “handicap”). The specified follow-up questionnaires addressed in particular changes in parameters (i.e., life style, diet and exercise habits, pharmacologic therapy, operations, and diseases) with a relevant impact on the main study outcomes. Furthermore, participants were provided with standardized diet records (Freiburger Nutrition Record, nutri-science, Hausach, Germany) that they had to conduct over 3 days at baseline and follow-up. The corresponding software used this list to calculate energy and macronutrient intake. Of importance, the primary investigator (CZ-R) checked all the questionnaires together with the participants to ensure consistency, completeness, and accuracy.

### Sample Size Calculation

The sample size of the study was powered on the initially intended primary study outcome “Average golf score of 5 rounds on an 18-hole course,” which unfortunately could not be addressed due to the temporary closure of all golf courses in our region.

### Statistical Procedures

We performed an intention-to-treat (ITT) analysis that included all participants initially assigned to the study arms (…“once randomized always analyzed”). In order to impute missing data, R statistics software (R Development Core Team, Vienna, Austria) in combination with Amelia II (Honaker et al., 2011) was used for the multiple imputation approach. The imputation was repeated 100 times using the full data set. Imputation diagnostic plots indicated that the imputation worked well for the outcomes addressed. After checking normal distribution, we applied paired *t*-tests to analyze within group changes over time (pre-post). ANCOVA that adjusted for baseline group differences of the corresponding outcome was applied to analyze time-group interactions (“effects”). Within- and between-imputation variance was calculated using the approach of Barnard and Rubin (1999). Due to the rather high number of missing values (each *n* = 9 in the WB-EMS and CG; Figure 2), we additionally applied per-protocol analyses that included all participants with complete MRI data sets for the main outcomes. We applied two-tailed tests and accepted significance at *p* < 0.05. We also calculated standardized mean difference (*d*´) to analyze effect sizes.

## Results

Reviewing the baseline characteristics displayed in Table 1, we observed no significant differences between the WB-EMS and CG.

**Table 1:** Baseline characteristics of the control group and WB-EMS group.

Variable	Control *n* = 27 MV ± SD	WB-EMS *n* = 27 MV ± SD
Age (years)	43.0 ± 13.4	42.7 ± 16.6
Body mass index (kg/m^2^)	26.7 ± 3.7	27.5 ± 4.7
Lean body mass (kg)^a^	66.0 ± 6.7	69.2 ± 10.6
Total body fat (%)^a^	22.7 ± 6.3	23.6 ± 8.5
Physical activity (score)^b^	3.5 ± 1.4	3.2 ±1.1
Handicap (score points)	18.4 ± 14.7	16.8 ± 13.7
History of golfing (years)	10 ± 6	11 ± 6
Golf frequency (sessions/week)	1.8 ± 1.3	2.1 ± 1.0
Other exercise (%)^c^	44	59
Leg press performance (N)	3729 ± 889	3581 ± 754
Orthopedic limitations (%)	48	48
Low back pain (LBP; %)^d^	93	89

a As determined by dual-segmental multi-frequency bio-impedance analysis.

b 1: very low to 7: very high (Kemmler et al., 2004b, 2014).

c ≤60 min/week.

d At least 1 day with LBP during the last week.

### Dropout and Attendance

Although we strictly implemented the COVID-19-related WB-EMS specification and visited participants at home or locations of their choice, seven participants of the WB-EMS group quit the intervention due to fear of infection. A further WB-EMS participant tested positive for COVID-19 also quit the intervention. Lastly, one participant of the WB-EMS group was unable to visit the 16-week FU assessment. In parallel, seven participants of the CG were unwilling to be assessed for follow-up due to fear of being infected during the FU tests; two further participants lost interest and could not be persuaded to attend the FU tests. Due to the possibility to make up a missed session, average WB-EMS attendance was close to the intended one session/week, only three WB-EMS participants did not attend all sessions. Also of importance, compliance with the WB-EMS protocol was recorded as high by the instructors, who documented an average impulse intensity of RPE 6.7 ± 0.5 over the last 12 weeks of the intervention period (…RPE 6–7 was intended).

### Main Outcomes

Volume-adjusted IMAT volume increased significantly in the CG (*p* = 0.040) and decreased non-significantly in the WB-EMS group (*p* = 0.215). Group differences for changes as determined by ITT, multiple imputation, and ANCOVA were significant (*p* = 0.028; effect size: *d*´ = 0.66; Table 2). This result was confirmed by the per-protocol analysis (*p* = 0.019).

**Table 2:** Baseline data and changes of study outcomes in the WB-EMS and control group.

	CG (*n* = 27) MV ± SD	WB-EMS (*n* = 27) MV ± SD	Difference MV (95% CI)	Value of *p*
IMAT volume of the mid-thigh (cm^3^)/intrafascial volume (cm^3^)				
Baseline	0.0566 ± 0.0255	0.0650 ± 0.0251	–	0.271
Changes	0.0041 ± 0.0095	−0.0025 ± 0.0104	0.0066 (0.0006–0.0126)	0.028
Muscle tissue volume of the mid-thigh (cm^3^)/intrafascial volume (cm^3^)				
Baseline	0.907 ± 0.039	0.896 ± 0.40	–	0.369
Changes	−0.0032 ± 0.0126	0.0059 ± 0.0134	0.0091 (0.0014–0.0167)	0.020
IMAT volume of the mid-thigh (cm^3^)				
Baseline	1434 ± 200	1459 ± 213	–	0.695
Changes	5.43 ± 19.20	−3.30 ± 20.24	8.73 (−2.75 to 20.21)	0.186
Muscle tissue volume of the mid-thigh (cm^3^)				
Baseline	1579 ± 192	1631 ± 251	–	0.447
Changes	−17.0 ± 86.0	22.6 ± 85.9	39.6 (−16.1 to 95.2)	0.154
Intrafascial volume of the mid-thigh (cm^3^)				
Baseline	1579 ± 192	1631 ± 251	–	0.447
Changes	−13.3 ± 86.3	16.4 ± 90.9	29.7 (−25.4 to 84.8)	0.266
Muscle tissue fat fraction (%)				
Baseline	4.31 ± 1.24	4.68 ± 1.15	–	0.299
Changes	0.019 ± 0.51	−0.073 ± 0.370	0.091 (−0.179 to 0.363)	0.491

Volume-adjusted muscle tissue volume decreased in the CG (*p* = 0.206) and increased significantly in the WB-EMS group (*p* = 0.032). Changes between CG and WB-EMS differ significantly applying ITT (*p* = 0.020; effect size: *d*´ = 0.70) or per-protocol analysis (*p* = 0.011).

### Explanatory Outcomes

IMAT increased non-significantly in the CG (*p* = 0.167) and decreased non-significantly in the WB-EMS group (*p* = 0.396). Group difference for changes in IMAT volume did not differ significantly (*p* = 0.186) when applying ITT (Table 2).

Muscle tissue volume decreased in the CG (*p* = 0.333) and increased in the WB-EMS group (*p* = 0.171) and increased in the WB-EMS group. The corresponding group difference was not significant (*p* = 0.154; Table 2).

Intrafascial volume decreased non-significantly in the CG (*p* = 0.438) and increased non-significantly in the WB-EMS group (*p* = 0.354). The corresponding difference between the groups was non-significant (ITT: *p* = 0.266; Table 2).

Muscle tissue fat fraction decreased slightly in the CG (*p* = 0.840) and increased in the WB-EMS group (*p* = 0.345). Corresponding group difference was non-significant (*p* = 0.491; Table 2).

### Confounding Parameters

Based on follow-up questionnaires, nutritional records and personal interviews with the participants at FU, we found no relevant changes in lifestyle, physical activity, exercise, or dietary habits. Further, we did not determine any changes or upcoming diseases or operations. Additionally, no changes in general medication were observed; however, there was a trend to a decrease in the acute intake of analgesics in the WB-EMS group. This might relate to a pronounced decrease (*p* = 0.080) in average LBP frequency in the WB-EMS group.

## Discussion

One main aim of the present project focuses on the effect of once 20 min/week WB-EMS, the standard protocol of commercial, non-medical WB-EMS in a cohort of moderately sportive men 18–70 years old. In a previous article of this project (Zink-Rückel et al., 2021), we reported the positive effects on once weekly WB-EMS on trunk (*p* = 0.001, *d*´ = 1.33) and leg extensor strength (*p* = 0.001, *d*´ = 0.94). However, apart from the simple proof of principle, certainly most welcome for the commercial WB-EMS community, the underlying mechanisms for these changes are of scientific interest.

In summary, we observed significant positive effects on muscle quality defined as fatty muscle infiltration as determined by 3D non-invasive MRI. In detail, our results show that volume-adjusted IMAT and muscle tissue volume were reduced significantly in the WB-EMS group compared with the CG. An increase of volume-adjusted IMAT reflects relative muscle tissue atrophy and vice versa, because our analysis approach divided the intrafascia volume into IMAT and muscle tissue. Although non-volume-adjusted parameters were not significant, they show a consistent picture of the training effects. Numerically, IMAT and muscle tissue fat fraction decreased and muscle volume increased in the training group with opposite effects in the control group.

The present study confirmed our results of a recent high-intensity resistance training (HIT-RT) approach with a cohort of older men with osteosarcopenia (Ghasemikaram et al., 2021). However, the latter study (Ghasemikaram et al., 2021) observed higher effects in particular on volume-adjusted IMAT^2^ (*d´* = 87) as determined by identical methods on the same 3-Tesla MRI scanner. However, apart from the much longer intervention period (16 weeks vs. 16 months), the present cohort was younger and more physically fit compared to the osteosarcopenic cohort of men 72 years and older (Kemmler et al., 2020; Ghasemikaram et al., 2021). Further, although speculative, we feel that the (effective^3^) training frequency of once per week 20 min might have been borderline low for this moderately sportive cohort of amateur golf players.

Two other studies using MRI (Marcus et al., 2010; Konopka et al., 2018) for assessing IMAT at the mid-thigh also confirmed our results. Albeit without control groups, Marcus et al. (2010) reported a significant decrease in IMAT after resistance and Konopka et al. (2018) after aerobic exercise. In contrast, Jacobs et al. (2014) could not detect a decrease in IMAT in older adults at risk of falling. Of course, it is difficult to compare these with our study. Apart from the exercise intervention (WB-EMS vs. predominately RT), participant age and status^4^ also differ between the studies. Moreover, Marcus et al. (2010) and Jacobs et al. (2014) analyzed single slices only and none of the three studies normalized IMAT area to (changes in) intrafascial area.

Some limitations might have affected the proper interpretation of our results. (1) Although our innovative MRI approach directly targets the quantitative assessment of fat infiltration from Dixon FF images (Chaudry et al., 2020), other studies used slightly different approaches, which complicates comparison of results across studies and calls for further standardization. (2) Unlike others (Ogawa et al., 2020), we also did not determine adipose tissue of individual thigh muscles. We justify this approach with the fact that WB-EMS application of the upper leg stimulates all the hamstring and quadriceps muscle groups simultaneously, although one may argue that superficial muscle groups might benefit more (Paillard, 2018). (3) Due to the high amount of lost-to-FU data that had to be imputed, we observed a high variation of the individual changes at least compared to the PP analysis that consistently revealed more significant effects. (4) We included male^5^ amateur golf players, which might not be the most relevant cohort for determining fatty muscle infiltration, however. We agree, but considering age, anthropometric and physical activity, the participants’ characteristics (Table 1) widely meet those of a non-athletic male population, so we feel that our results can be widely generalized. Nevertheless, our male cohort was very heterogeneous with respect to age. Therefore, we compare participants 45 years and younger vs. participants older than 45 years for our main outcomes in order to check for an age-dependent relationship. In summary, we do not observe relevant differences between the groups; however, the statistical power to address this issue was limited. (5) Another minor bias was that our sample size analysis was based on the initial primary outcome average “golf score,” which was lost. However, as expected, the statistical power was high enough to address MRI data successfully.

In summary, we provide further evidence for a positive effect of WB-EMS on muscle quality as determined by MRI while assessing means of fatty muscle infiltration. Of importance however, future studies should further verify this result for more vulnerable cohorts with sarcopenia, sarcopenic obesity, or osteosarcopenia, i.e., people unable or unmotivated to start conventional muscle strengthening exercise.

## Data Availability Statement

The raw data supporting the conclusions of this article will be made available by the authors, without undue reservation.

## Ethics Statement

The studies involving human participants were reviewed and approved by Ethikkkommission der Friedrich-Alexander-UniversitätErlangen-Nürnberg, Krankenhausstrasse 12, 91052 Erlangen, Germany (number 377_19b). The patients/participants provided their written informed consent to participate in this study.

## Author Contributions

CZ-R, OC, MG, MK, KE, MU, and WK contributed to conception and design of the study. OC, KE, and MU organized the MRI assessments and analysis. MK performed the statistical analysis. CZ-R and WK wrote the first draft of the manuscript. KE and OC wrote sections of the manuscript. All authors contributed to manuscript revision, read, and approved the submitted version.

### Conflict of Interest

The authors declare that the research was conducted in the absence of any commercial or financial relationships that could be construed as a potential conflict of interest.

## Footnotes

^1^The difference in training frequency can be considered as the main difference between the commercial WB-EMS setting and the protocol of scientific WB-EMS studies. Impulse parameters were closely comparable (bipolar, 85 Hz, 350 Î¼s, 4–6 s of impulse – 4 s of impulse break, impulse intensity 5–7 on Borg CR 10).

^2^In this study, we applied the exactly same procedure at the same MRI scanner.

^3^As given attendance rate was close to 100%.

^4^Healthy 18–70 years old men vs. predominately older cohorts (Marcus et al., 2010; Konopka et al., 2018) or “fallers” (Jacobs et al., 2014).

^5^We focus on one gender because [although not undisputed (Staron et al., 1994; Roth et al., 2000)] there is some evidence in humans for gender differences in the muscle mass response to resistance training (Hakkinen et al., 1998; Ivey et al., 2000).
